# Transcriptional activity and variation analysis of genes critical for long-chain fatty acid (C≥16) elongation and desaturation in Pekin ducks

**DOI:** 10.1016/j.psj.2024.104437

**Published:** 2024-10-18

**Authors:** Dandan Sun, Yongtong Liu, Xiaoqin Li, Mengqi Ge, Meiyi Zhu, Yuqin He, Zhuocheng Hou

**Affiliations:** College of Animal Science and Technology, China Agricultural University, Beijing 100193, China

**Keywords:** Duck, Fatty acid, Transcription factors, Variation, Molecular regulation

## Abstract

To deepen our understanding of long-chain fatty acid carbon chain elongation and desaturation in ducks, this study systematically analyzed the transcriptional activities of key gene promoters, including *ELOVL*s, *FADS*s, and *SCD*s. Predictive modeling coupled with statistical analysis revealed a prevalence of binding motifs for transcription factors, particularly those associated with *Sp1, NF-1*, and *C/EBPalpha*. Moreover, variation analysis of resequencing data from both wild and domestic ducks, specifically mallards and Pekin ducks, informed targeted mutagenesis within the core promoter regions of *ELOVL2, ELOVL5*, and *ELOVL6*. Notably, mutations at positions -56 G>C in *ELOVL2*, -52 T>C in *ELOVL5*, and -513 T>C in *ELOVL6* significantly diminished transcriptional activity. These findings substantially enhance our understanding of the molecular mechanisms regulating the biosynthesis of long-chain fatty acids in ducks and support future genetic selection initiatives aimed at developing Pekin duck breeds with enhanced nutritional value.

## Introduction

Lipids are apolar products, primarily classified as fatty acids and their derivatives like waxes, triglycerides, phospholipids, and other related molecules, which are crucial for energy storage in organisms, maintaining the normal structure of membranes, and the synthesis of hormones (Dickschat, 2017). The glycerol component in lipids is uniform and structurally simple, whereas fatty acids are diverse in type and function, thereby determining the nature and characteristics of lipids, and they are crucial for the health and metabolic processes of organisms. Fatty acids are categorized into saturated and unsaturated fatty acids, with the former characterized by carbon-carbon single bonds, and the latter containing one or more double bonds. Polyunsaturated fatty acids (PUFAs) (C≥16) are particularly important for human health, as they regulate cholesterol levels, influence cardiovascular health, and are crucial for growth and development.

The human body has limited efficiency in synthesizing PUFAs, primarily obtaining them through the consumption of meat, eggs, and dairy products. Current strategies for manipulating the fatty acid composition in animals involve adjusting dietary components and rearing conditions to enhance production performance and product quality (Valentini et al., 2020). However, this approach does not fully exploit the genetic potential of animals in PUFA synthesis. Studies have shown that poultry, particularly ducks, have a distinct advantage in terms of PUFA content and proportion (Onk et al., 2019), making them an ideal source for human consumption. Research into the processes of carbon chain elongation and desaturation of long-chain fatty acids may yield substantial benefits for the enhancement of poultry product quality, as well as for meeting human nutritional demands and improving health outcomes.

In the synthesis of PUFAs, key gene families such as *ELOVL, SCD*, and *FADS* play essential roles. *ELOVL* genes are responsible for the elongation of PUFA chains, while *FADS* and *SCD* genes catalyze the desaturation process. *SCD* and *FADS* are rate-limiting enzymes in the desaturation process, with *SCD* converting saturated fatty acids (SFAs) to monounsaturated fatty acids (MUFAs), and *FADS* converting MUFAs to PUFAs. The interplay between *ELOVL, SCD*, and *FADS* in the PUFAs synthesis pathway is critical, and their expression levels are significant determinants of the synthesis and metabolism of PUFAs. Investigating the regulatory mechanisms affecting the expression of *ELOVL, SCD*, and *FADS* gene families is key to improving the synthesis of PUFAs.

A burgeoning body of research has underscored the significant influence that regulatory regions exert on the manifestation of phenotypic traits. Specifically, the use of specific single nucleotide polymorphisms (SNPs) within promoter regions has facilitated the development of efficient and accurate molecular marker-assisted breeding techniques (Zhao et al., 2022). Promoter sequences modulate transcriptional initiation via interactions with transcription factors and regulatory elements, thereby affecting protein synthesis dynamics. Investigating the promoter functions of genes such as *ELOVL, SCD*, and *FADS* in duck PUFA synthesis, along with the identification of crucial motifs, is essential for elucidating the mechanisms of gene expression and its transcriptional control.

Breeds/strains impacts fatty acid composition, with gene expression for polyunsaturated fatty acid synthesis varying across duck breeds. The enhanced fatty acid profile in crossbred strains' breast muscle correlates with increased expression of genes including *SCD1, FADS2, ELOVL2*, and *ELOVL5* (Zhang et al., 2022). The Pekin duck is a world-standard breed that stands out for its superior meat texture, and its genetic diversity is valuable for crossbreeding to improve other duck breeds. Our study has pinpointed the key functional areas within the promoters of the *ELOVL, SCD*, and *FADS* gene families, which are crucial for the synthesis of PUFAs in Pekin ducks. We have predicted the Transcription factors (**TFs**) involved in these core promoter functional regions and discovered that certain SNPs within these regions exhibit differential activity in vitro. This generates robust empirical evidence to undergird future scholarly endeavors investigating the influence of genetic variations within the upstream promoter regulatory domains on the biosynthetic pathways of PUFAs in avian species, specifically ducks.

## Materials and methods

### *Ethics statement*

This experiment was approved by the Animal Care and Use Committee of China Agricultural University (permit number: SYXK 2007–0023) and was conducted according to the Guidelines of Ministry of Science and Technology of the People's Republic of China (Beijing, China).

### *Construction of luciferase vector, cell culture and transfection, dual-luciferase reporter gene assay*

Genomic DNA was isolated from Pekin duck blood samples. The primer design (Table 1), the enzymatic digestion of plasmid, the purification and recovery of the target fragments, the ligation and transformation of recombinant plasmids, the extraction of plasmid DNA, cell culture, and transfection, as well as the Dual-Luciferase Reporter gene assay were performed as previously described (Liu et al., 2023). To ensure the accuracy of the sequences, all plasmids underwent Sanger sequencing prior to their utilization.

**Table 1:** Design of primers for amplifying duck *ELOVL*s, *SCD*s, and *FADS*s gene promoter and for mutagenesis of *ELOVL2, ELOVL5*, and *ELOVL6* promoter recombinant plasmids.

Gene ID	Primer Name	Primer Sequences
ENSAPLG00020001674	ELOVL1(-2234)F	5′-ggcctaactggccGGTACC GATGTGCTGGAAAACCCCTG-3′
	ELOVL1(-1633)F	5′-ggcctaactggccGGTACC GCAGTGCAAGATGTAGGTCTG-3′
	ELOVL1(-1274)F	5′-ggcctaactggccGGTACC CAGTTCCTGCTGCCTTCTTC-3′
	ELOVL1(-987)F	5′-ggcctaactggccGGTACC CGGGCTGAAGGATAGATGCT-3′
	ELOVL1(-538)F	5′-ggcctaactggccGGTACC GTGGTCATCAGCCTTCTCCT-3′
	ELOVL1(-135)F	5′-ggcctaactggccGGTACC CTGGTGTAGAACTGGCTGGA-3′
	ELOVL1(+82)R	5′-gaggccagatcttgatatcCTCGAGG CAAACACCCACCTGCTTTCT-3′
ENSAPLG00020012020	ELOVL2(-2245)F	5′-ggcctaactggccGGTACC GCAGAGGAGTTGGACCAGAT-3′
	ELOVL2(-1901)F	5′-ggcctaactggccGGTACC GGGATCCAACAACATGTGCA-3′
	ELOVL2(-1429)F	5′-ggcctaactggccGGTACC GGTTCTTTGCCAGTCACCTT-3′
	ELOVL2(-671)F	5′-ggcctaactggccGGTACC TCTCCAGACTGAACAACCCC-3′
	ELOVL2(-329)F	5′-ggcctaactggccGGTACC ACAGTCTGTTCTCCGAGCTC-3′
	ELOVL2(-126)F	5′-ggcctaactggccGGTACC AGAAGGGCATTTTCGGCAAG-3′
	ELOVL2(+295)R	5′-gaggccagatcttgatatcCTCGAGG CCTCCACTCATCCCTCCCT-3′
ENSAPLG00020013672	ELOVL3(-1757)F	5′-ggcctaactggccggtaGGTACC TGGGAAACAAGTCATGTCCA-3′
	ELOVL3(-1419)F	5′-ggcctaactggccggtaGGTACC CAGGGCAATTCCTCTAGCAT-3′
	ELOVL3(-1150)F	5′-ggcctaactggccggtaGGTACC CCCTGGGACAGCTCACAGTA-3′
	ELOVL3(-667)F	5′-ggcctaactggccggtaGGTACC CACATCCTTCCATGCCACAT-3′
	ELOVL3(-473)F	5′-ggcctaactggccggtaGGTACC AAAGATGATGCCCAAATTGC-3′
	ELOVL3(-290)F	5′-ggcctaactggccggtaGGTACC GATAACTGGGTGCAGGTTCC-3′
	ELOVL3(+167)R	5′-cgaggccagatcttgatatcCTCGAGG CTCCGCTCCACCTCATACTC-3′
ENSAPLG00020009654	ELOVL4(-2236)F	5′-ggcctaactggccGGTACC GGGATGTGTTTGGAAGCCTG-3′
	ELOVL4(-1872)F	5′-ggcctaactggccGGTACC TTATTGGTGTTAGTTGGGCAAAA-3′
	ELOVL4(-1396)F	5′-ggcctaactggccGGTACC GCACAGGAACCACAGCTTAC-3′
	ELOVL4(-1067)F	5′-ggcctaactggccGGTACC CCCAGAGTCAGTGATCCCTG-3′
	ELOVL4(-617)F	5′-ggcctaactggccGGTACC AGGGCTTGTCTGGGTTGATC-3′
	ELOVL4(-227)F	5′-ggcctaactggccGGTACC GTGAACACCTGAGGCGAAG-3′
	ELOVL4(+121)R	5′-gaggccagatcttgatatcCTCGAGG CCTTGTAGAGCTGGGGATGT-3′
ENSAPLG00020012876	ELOVL5(-1989)F	5′-ggcctaactggccGGTACC GGAGGTTGAAGAGCTTATCTTTTTCCTAG-3′
	ELOVL5(-1273)F	5′-ggcctaactggccGGTACC CCTTGAACACCTCCAGGGATG-3′
	ELOVL5(-731)F	5′-ggcctaactggccGGTACC AGTCTATCTTTAGTACCCAGCTGTG-3′
	ELOVL5(-392)F	5′-ggcctaactggccGGTACC GCACTTTCCCCCAGATCCTTTC-3′
	ELOVL5(-166)F	5′-ggcctaactggccGGTACC GCAGGCAGTGTTTTTCCTTTGAG-3′
	ELOVL5(+69)R	5′-gaggccagatcttgatatcCTCGAGG GGAAAGAAAGCAATTCTACCTGTAG-3′
ENSAPLG00020017528	ELOVL6(-1977)F	5′-ggcctaactggccGGTACC ACTGGTGATGAGTGGTGTTCC-3′
	ELOVL6(-1646)F	5′-ggcctaactggccGGTACC AGCTTAACAACCTGTGCTTGC-3′
	ELOVL6(-1299)F	5′-ggcctaactggccGGTACC AGGAGCACTCCCATGTCTCA-3′
	ELOVL6(-1149)F	5′-ggcctaactggccGGTACC GGACATCTGGAAGACCACTGT-3′
	ELOVL6(-667)F	5′-ggcctaactggccGGTACC GCTGTTGATATCCCACGACCT-3′
	ELOVL6(-297)F	5′-ggcctaactggccGGTACC CTGAACGGGGACACCAGAA-3′
	ELOVL6(+46)R	5′-gaggccagatcttgatatcCTCGAGG GTCTCCTCCTCTTCTGCAG-3′
ENSAPLG00020003872	ELOVL7(-2291)F	5′-ggcctaactggccGGTACC GGAAAGCAAACAAGACCTGGT-3′
	ELOVL7(-1840)F	5′-ggcctaactggccGGTACC GGACCTGAGCCACCACTTAT-3′
	ELOVL7(-1348)F	5′-ggcctaactggccGGTACC CAGTGTCCCATTTTGCCACA-3′
	ELOVL7(-940)F	5′-ggcctaactggccGGTACC GATGCCCTCTCAAAAGTGCC-3′
	ELOVL7(-527)F	5′-ggcctaactggccGGTACC ACCCTCTCCTTCCTCCTAGA-3′
	ELOVL7(-232)F	5′-ggcctaactggccGGTACC GCTTGAGGTATTGGGCTGGA-3′
	ELOVL7(+216)R	5′-gaggccagatcttgatatcCTCGAGG AAGCAGTGATTTTGGGGCAG-3′
ENSAPLG00020005032	FADS1(-2124)F	5′-ggcctaactggccGGTACC AGGACATTGAACCCCTCGCT-3′
	FADS1(-1825)F	5′-ggcctaactggccGGTACC TTCCTAAGGCTCAATCTG-3′
	FADS1(-1349)F	5′-ggcctaactggccGGTACC GGCTGGAAAGTCTCAGTGTAGT-3′
	FADS1(-1079)F	5′-ggcctaactggccGGTACC CTGTTTGCTGGGGGTGTTTG-3′
	FADS1(-681)F	5′-ggcctaactggccGGTACC ACCCAGCCTCTGTCCTTTCC-3′
	FADS1(-390)F	5′-ggcctaactggccGGTACC AGCCAACCAGTCCTTAGGCAA-3′
	FADS1(+150)R	5′-gaggccagatcttgatatcCTCGAGG CCGGGGATACCTCGTCATGC-3′
ENSAPLG00020004999	FADS2(-2187)F	5′-ggcctaactggccGGTACC GCTTACCCTGCTGAGTACCA-3′
	FADS2(-1811)F	5′-ggcctaactggccGGTACC CTCAGCCCACCAGTCTAACC-3′
	FADS2(-1488)F	5′-ggcctaactggccGGTACC GGTTGAAGCACACTTGATGAGG-3′
	FADS2(-1059)F	5′-ggcctaactggccGGTACC GACAGCACAAGTGGGATTGGA-3′
	FADS2(-673)F	5′-ggcctaactggccGGTACC GTAGGGAGCGTAGGTGGGAA-3′
	FADS2(-133)F	5′-ggcctaactggccGGTACC AGCAAACAGCCACAAGTAAGG-3′
	FADS2(+35)R	5′-gaggccagatcttgatatcCTCGAGG GTCGTTAGGTTATGCAAGGCG-3′
ENSAPLG00030003121	FADS6(-1960)F	5′-ggcctaactggccGGTACC CACCCAACCCGAGTAACAAC-3′
	FADS6(-1654)F	5′-ggcctaactggccGGTACC GCCTCCTACTCCTCCTGTG-3′
	FADS6(-1313)F	5′-ggcctaactggccGGTACC CTCATTTGGGGCTTGGACAC-3′
	FADS6(-985)F	5′-ggcctaactggccGGTACC TTCCCTCCTTAACGAGCTGG-3′
	FADS6(-633)F	5′-ggcctaactggccGGTACC GTTTGCCTCCCAGTCGCT-3′
	FADS6(-205)F	5′-ggcctaactggccGGTACC TCCCTCTTTCACCATCCCAC-3′
	FADS6(+42)R	5′-gaggccagatcttgatatcCTCGAGG AGAGGGAGGAGGGAACGAG-3′
ENSAPLG00020013562	SCD1(-2281)F	5′-ggcctaactggccGGTACC CCCTTAGCCATGCTCTGCTT-3′
	SCD1(-1799)F	5′-ggcctaactggccGGTACC CCTTTCTGACTCATGCCAGC-3′
	SCD1(-1531)F	5′-ggcctaactggccGGTACC TCACTTTTGACAGCTGCCAC-3′
	SCD1(-1240)F	5′-ggcctaactggccGGTACC ACTAAAGCACCTCAGGCCTT-3′
	SCD1(-739)F	5′-ggcctaactggccGGTACC GGAGCTGTTTTGGGGTGAAG-3′
	SCD1(-301)F	5′-ggcctaactggccGGTACC TTCCTCAGCATCCTTCCTCC-3′
	SCD1(+372)R	5′-gaggccagatcttgatatcCTCGAGG GCAGGCTTGGATCGATGTTT-3′
ENSAPLG00020004606	SCD5(-2115)F	5′-ggcctaactggccGGTACC CCATGAGCTTCAGAGACGGA-3′
	SCD5(-1721)F	5′-ggcctaactggccGGTACC GTCCCCACAAATCATCTGCC-3′
	SCD5(-1368)F	5′-ggcctaactggccGGTACC TTCTTTTGCTGTGGAGGCTG-3′
	SCD5(-1001)F	5′-ggcctaactggccGGTACC ATCCCATTTCGTGTGTGCTG-3′
	SCD5(-679)F	5′-ggcctaactggccGGTACC CCCCACTCACACATCACAAG-3′
	SCD5(-236)F	5′-ggcctaactggccGGTACC GTGAGGGGTGATTGCTGAAC-3′
	SCD5(+11)R	5′-gaggccagatcttgatatcCTCGAGG CCTCACAGTAGTCCCCTCAC-3′
ENSAPLG00020012020	*ELOVL2*-MU-56G>C-F	5′-CTTTGTTTcCACTCGGAACAAAACCCGCAGCT-3′
	*ELOVL2*-MU-56G>C-R	5′-TCCGAGTGgAAACAAAGCATTACATCTCCTATAAAAAA-3′
ENSAPLG00020012876	*ELOVL5*-MU-52T>C-F	5′-TTCTCACCcGAAAGCAAACAAGAAGACAGGAGG-3′
	*ELOVL5*-MU-52T>C-R	5′-TTGCTTTCgGGTGAGAAAGACCAACCAAGGGG-3′
ENSAPLG00020017528	*ELOVL6*-MU-513T>C-F	5′-TAAGGACAcGTTGAGTGCCCTATGAGGAAAATT-3′
	*ELOVL6*-MU-513T>C-R	5′-CACTCAACgTGTCCTTAATTTTGTCTTTCCTGC-3′

Notes. The designed primer is based on duck genome assembly ASM874695v1 (Ensembl Release 106). Only the coordinates of the *FADS6* core promoter region correspond to the assembly of duck genome GCF_015476345.1 (Ensembl Release 104), and the sequence before the space denotes the restriction enzyme sites.

### *Construction of site-directed mutagenesis plasmids*

The previously amplified core promoter region sequences of duck *ELOVL2, ELOVL5*, and *ELOVL6* were sequenced. Single-point mutation primers were designed at the corresponding SNP sites using the online platform CE Design (https://crm.vazyme.com/cetool/singlepoint.html) (Table 1). Site-directed mutagenesis was performed on the pGL4.10 recombinant plasmids, which had the target gene core promoter regions inserted. The Mut Express MultiS Fast Mutagenesis Kit V2 (Vazyme, China) was employed to conduct PCR using the original recombinant plasmids *ELOVL2*-126, *ELOVL5*-166, and *ELOVL6*-667 as templates. *Dpn*I was added to the amplified products to digest and remove the methylated template DNA. The linear DNA was circularized in vitro as per the protocol, then transformed into DH5α cells (Vazyme, China) and purified through single colony selection. The successfully sequenced target site-directed mutants were extracted using the EndoFree Midi Plasmid Kit (TIANGEN, China).

### *Statistical analysis*

The protocol for transfecting each recombinant plasmid was carried out in triplicate, and the luminescence detection assay was also performed triplicately for every individual well-containing cell lysate. Data were analyzed using SPSS 27.0 software. Experiments included three biological and technical replicates each. The *t*-tests and Duncan's tests following ANOVA were used for statistical comparisons. Results were shown as mean ± standard deviation (SD), with *P*<0.05 indicating significance.

### *Forecasting transcription factor binding in the core promoter segments*

The interaction of transcription factors with the core promoter areas of genes involved in duck PUFA biosynthesis was anticipated through the web-based tool AliBaba2.1 (http://gene-regulation.com/pub/programs/alibaba2/index.html).

## Results and discussion

**Fig. 1:**
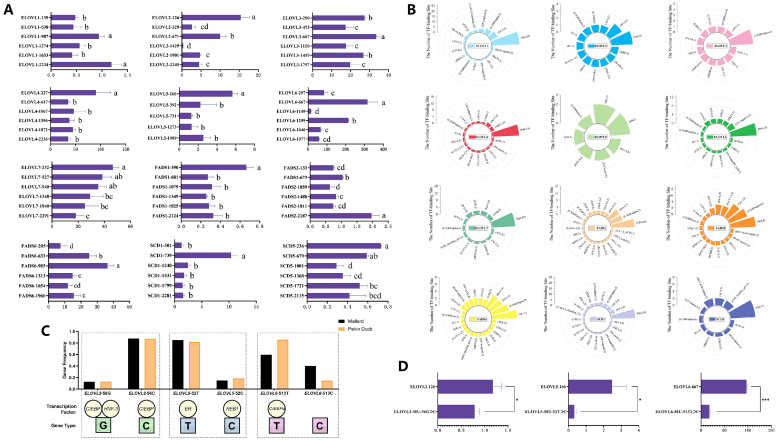
(A) Detection of relative luciferase activity in different truncated fragments of duck *ELOVL*s, *FADS*s, and *SCD*s gene promoters. The ordinate represents the gene name and the distance from the TSS. For example, *ELOVL1*-135 indicates that the gene name is *ELOVL1*, and the number 135 represents the region from 135 base pairs upstream of the TSS; the abscissa represents the relative luciferase values obtained using the empty pGL4.10 vector as a control. (B) Statistics of TF binding in the core promoter regions of duck PUFA-related gene families. Each bar represents a TF, with the bar length indicating the number of TFBSs, and the bar annotation reads: (TF name, number of TFBSs). (C) Frequency of SNPs within the key functional areas within the promoters of *ELOVL2, ELOVL5*, and *ELOVL6* and the predicted binding of TFs in mallard and Pekin duck. (D) Detection of relative luciferase values for the original core promoter region plasmids and site-mutated plasmids of duck *ELOVL2, ELOVL5*, and *ELOVL6*. The ordinate represents the gene name and the distance from the TSS or the specific point mutation position relative to the TSS. The abscissa represents the relative luciferase values obtained using the pGL4.10 vector as a control.

We systematically analyzed the transcriptional activities of the *ELOVL*s (*ELOVL1, -2, -3, -4, -5, -6*, and *-7*), *FADS*s (*FADS1, -2*, and *-6*), and *SCD*s (*SCD1* and *SCD5*) genes within the first 2000 bp upstream of the transcription initiation sites. The core region is defined as the segment that, upon addition, results in the highest promoter activity. The results revealed that the core promoter regions for the aforementioned genes are located at -539/-987 bp (Chromosomal Location: 21749916/21750364), -1/-126 bp (Chromosomal Location: 94618999/94619124), -474/-667 bp (Chromosomal Location: 20800779/20800972), -1/-227 bp (Chromosomal Location: 35536081/35536307), -1/-166 bp (Chromosomal Location: 25531134/25531299), -298/-667 bp (Chromosomal Location: 4352282/4352651), -1/-232 bp (Chromosomal Location: 20136087/20136318), -1/-390 bp (Chromosomal Location: 54137281/54137670), -134/-673 bp (Chromosomal Location: 54128619/54129158), -634/-985 bp (Chromosomal Location: 9474778/9475129), -302/-739 bp (Chromosomal Location: 20086617/20087054), and -1/-236 bp (Chromosomal Location: 44900019/44900254) upstream of the transcription start site (TSS), respectively (Fig. 1A). Additionally, the relative luciferase activity levels of various excised segments situated within the confines of the promoter regions of these genes suggest the presence of regulatory domains that either enhance or repress transcriptional activity. For instance, enhancer structures are found within the sequences -539bp to -987bp upstream of *ELOVL1*, -330bp to -671bp upstream of *ELOVL2*, and -1150bp to -1299bp upstream of *ELOVL6*, while suppressor structures are located in the sequences -1275bp to -1633bp upstream of *ELOVL1*, -127bp to -329bp upstream of *ELOVL2*, and -668 bp to -1149 bp upstream of *ELOVL6*. These regulatory regions may significantly influence the transcriptional levels of the genes.

We conducted an analysis of truncated promoter activity in chicken and duck homologs associated with PUFA synthesis (Liu et al., 2023). Most related genes, including *ELOVLs (ELOVL2, -3, -4, -6, -7), FADS6*, and *SCD1*, exhibited comparable promoter activity between species. Chicken promoters for *ELOVL1, ELOVL5, FADS1, FADS2*, and *SCD5* generally displayed higher activity, except for duck *FADS6*. Activity trends in truncation fragments diverged only for *ELOVL5, FADS6*, and *SCD5*. Core promoter regions of *ELOVLs (ELOVL1, -2, -3, -4, -6, -7), FADS1, FADS2*, and *SCD1* maintained similar distances from the TSS within 1 Kb in both species, highlighting regulatory region conservation and divergence.

Open chromatin regions are integral to gene expression control. Core promoter locations identified via Dual-Luciferase Reporter Assay partially correspond to, but are not fully consistent with, predicted positions. Correlation with ATAC-seq data in ducks (Zhu et al., 2021) shows that most determined core promoters coincide with ATAC peaks located within 1kb proximity to the TSS, suggesting an open chromatin state for *ELOVLs (ELOVL1, -2, -4, -6, -7), FADSs (FADS2, -6)*, and *SCDs* (*SCD1*, -*5)*. In contrast, *ELOVL3, ELOVL5*, and *FADS1* core promoters are adjacent to ATAC peaks within a 500 bp range near the TSS, potentially due to transient signals in ATAC-seq (Liu et al., 2023). This study refines ATAC-seq precision and provides a foundation for exploring gene expression regulatory mechanisms.

Transcription factors govern gene expression and engage in regulatory networks, which are crucial for adipogenesis. This study predicts TFs associated with promoter core sequences and statistically evaluates binding site frequencies (Fig. 1B). Results suggest that duck gene promoters predominantly associate with TFs including *Sp1, C/EBPα, NF-1, Oct-1*, and *TBP*.

SNPs in core promoter regions of *ELOVL2, ELOVL5*, and *ELOVL6*, which may affect transcription factor binding (Fig. 1C), were targeted for mutagenesis based on luciferase assays and duck resequencing data (Zhu et al., 2021). The results showed that compared to the wild-type *ELOVL2*-126, *ELOVL5*-166, and *ELOVL6*-667, the mutations -56G>C in *ELOVL2*, -52T>C in *ELOVL5*, and -513T>C in *ELOVL6* significantly reduced transcriptional activity (Fig. 1D), indicating that the SNP variations present in the core promoter regions exhibit differential activity in vitro.

The predictive results suggest that TFs with a propensity for high-frequency association with the core promoter elements of genes involved in PUFA synthesis in ducks may be integral to the modulation of adipogenesis. Comparative analysis of TFs binding to the core promoters of these genes has been conducted between chickens and ducks (Liu et al., 2023). Firstly, this analysis revealed a consistency in the top three TFs predicted to bind to the core promoters across both species: *Sp1, C/EBPalpha*, and *NF-1*. These TFs are capable of modulating the metabolic pathways of fatty acids (Linsheng Gui, 2021). We propose the hypothesis that these three prevalent TFs exert a substantial influence on the regulation of fatty acid synthesis in poultry, potentially affecting the composition and ratio of fatty acids. Subsequent studies can proceed on two fronts: first, by experimentally validating the interaction and regulatory effects of these TFs with promoters; and second, by constructing gain-of-function and loss-of-function models of the TFs to confirm their impact on fatty acid synthesis. Secondly, there are differences in the binding frequency of some TFs between ducks and chickens, such as *TBP*, which has a higher frequency in ducks (14 motifs) than in chickens (2 motifs). *TBP* can modulate various cellular processes associated with lipogenesis (Fan et al., 2017). Some TFs are found only in the predicted results for ducks or chickens. The binding patterns indicate ducks may inherently have a richer PUFA profile than chickens. More research is essential to verify the influence of these factors on duck genes for PUFA synthesis, affecting gene activity and fatty acid levels.

Compared to mallards, Pekin ducks, which have undergone intense artificial selection, are known for their rapid growth rate and superior fat deposition ability. This selective pressure has led to a wealth of genetic variations within the Pekin duck genome, which are associated with economically significant traits. Studies have found that a high proportion of significant SNPs between mallards and domestic ducks are located in regulatory or non-coding regions, and their mutation rates are much higher than those in the coding areas (Zhu et al., 2021). Our site-directed mutagenesis experiments in core promoter regions revealed that the high-activity advantageous SNP *ELOVL6*-513T>C is much more prevalent in the Pekin duck population than in mallard, and TF prediction identified differences in the *CEBPA* binding site associated with this SNP. The high-activity advantageous SNPs *ELOVL2*-56G>C and *ELOVL5*-52T>C are fixed in Pekin ducks, and the impact of these SNPs on promoter activity may be due to changes in the binding sites of different TFs. These SNPs may be important factors contributing to the differences in PUFA synthesis content and efficiency between mallards and Pekin ducks. Regulatory region SNPs, and their combinations, may affect gene expression and phenotypes through changes in TF binding activity (Chen et al., 2020). Integrating synergistic combinations of highly active beneficial SNPs into molecular genetic enhancement strategies is likely to be of significant value in directing the selective breeding of Pekin ducks. This approach aims to achieve superior quality, marked by elevated levels and proportions of PUFAs. Therefore, further experiments are needed to investigate the mechanisms of action of these SNP combinations.

This study has identified the core functional regulatory regions within the promoter of key gene families that influence the elongation of carbon chains and the formation of double bonds in long-chain fatty acids in ducks, such as *ELOVL, FADS*, and *SCD*, and predicted the TFs in these regions. Additionally, it analyzed the variation information in these key regions between mallards and Pekin ducks, obtaining data on high-activity advantageous SNPs in the *ELOVL2, ELOVL5*, and *ELOVL6* core promoter regions of Pekin ducks. The study's findings contribute a robust dataset that serves as a foundational resource for subsequent investigations into the molecular underpinnings of PUFA enrichment in Pekin ducks. Furthermore, these findings establish a solid framework for investigating molecular markers associated with high PUFA content within the Pekin duck population.

## Disclosures

The authors declare that they have no known competing financial interests or personal relationships that could appear to influence the work reported in this paper.
